# Chinese patent medicine (Jinlong Capsule) for gastric cancer: Protocol for a systematic review and meta-analysis

**DOI:** 10.1097/MD.0000000000020532

**Published:** 2020-06-05

**Authors:** Jianwei Li, Bin Han, Guangzong Sun, Zhong Zheng, Ying Mu, Jingxia Chi

**Affiliations:** a Department of Gastroenterology; b Infection Control Office; c Department of Emergency, People's Hospital of Weifang Binhai Economic and Technological Development Zone; d Department of Gastroenterology, Weifang People's Hospital, Weifang; e Department of Gastroenterology, Liaocheng People's Hospital, Liaocheng; f Quality Management Office, People's Hospital of Weifang Binhai Economic and Technological Development Zone, Weifang, Shandong Province, China.

**Keywords:** efficacy, gastric cancer, Jinlong Capsule, meta–analysis, safety

## Abstract

**Background:**

JLC has been widely applied as a promising adjunctive drug for GC. However, the exact effects and safety of JLC have yet to be systematically investigated. We aimed to summarize the efficacy and safety of JLC for the treatment of advanced GC through the meta-analysis, in order to provide scientific reference for the design of future clinical trials.

**Methods:**

The protocol followed Preferred Reporting Items for Systematic Reviews and Meta-Analyses Protocols. Relevant randomized controlled trials were searched from Cochrane Library, PubMed, Web of Science (WOS), Excerpt Medica Database (Embase), Chinese Biomedical Literature Database (CBM), China National Knowledge Infrastructure (CNKI), China Scientific Journal Database (VIP), and Wanfang Database. Papers in English or Chinese published from their inception to January 2020 will be included without any restrictions.

Study selection and data extraction will be performed independently by 2 investigators. The clinical outcomes including overall response rate, complete response rate, overall survival, Disease-free survival, quality of life (QoL), immune function, and adverse events, were systematically evaluated. Review Manager 5.3 and Stata 14.0 were used for data analysis, and the quality of the studies was also evaluated.

**Results and conclusion:**

The findings of this research will be published in a peer-reviewed journal, and provide more evidence-based guidance in clinical practice.

**International Platform of Registered Systematic Review and Meta-Analysis Protocols (INPLASY) registration number:**

INPLASY202040105. URL: https://inplasy.com/inplasy-2020–4–0105/

## 1 Introduction

Gastric cancer (GC) is the third leading cause of cancer-related death and caused 782,685 deaths worldwide in 2018.^[1]^ Currently, the incidence of GC has significantly increased, with about 1.03 million new cases every year.^[1]^ Among them, 40% of newly diagnosed patients and 50% of death are from China.^[2]^ The etiology of GC is still unclear, with possible factors including regional environment, micronutrient deficiency, diet habits, obesity, work pressure, helicobacter pylori infection, and genetic factors.^[3,4]^ Despite the improvement of diagnostic and therapeutic methods in the past decades, the prognosis of GC remains unsatisfactory, with a median survival time of 8 to 11 months (5-year survival rate <30%).^[4]^

The first choice of the treatment for GC is systematic chemotherapy, radiotherapy, and palliative surgery. However, current treatment methods for GC only has a modest survival benefit and the disadvantages of radiochemotherapy such as drug resistance and toxic side effects have become a substantial burden on GC patients.^[5]^ On the other hand, the treatment of GC in the East and West is not exactly the same.^[6–10]^ Some researchers indicated that the combination of Chinese and Western medicine for GC may be the potential trend of clinical treatment development in future.^[8–11]^ Through the nationwide survey in China, the study indicates that 24.8% of the cancer patients used both Chinese patent and western anti-cancer medicines.^[5]^

Jinlong Capsule (JLC) is a Chinese patent medicine, which contains at least seventeen types of amino acids including arginine, histidine, aspartic acid, serine, glycine, etc and is extracted by the process of biochemical separation and modern cryogenic technologies from 3 fresh animals including Bungarus, Agkistrodon, and Gecko.^[12–18]^ JLC was manufactured by Beijing Jiansheng Pharmaceutical Co, Ltd. The Quality Standards of JLC have been approved by Chinese State Food and Drug Administration (SFDA), and granted the Manufacturing Approve Number accordingly (Z10980041). It was selected as "Chinese characteristic medicine” by the National Pharmacopoeia Committee in 2001.

**Figure 1:**
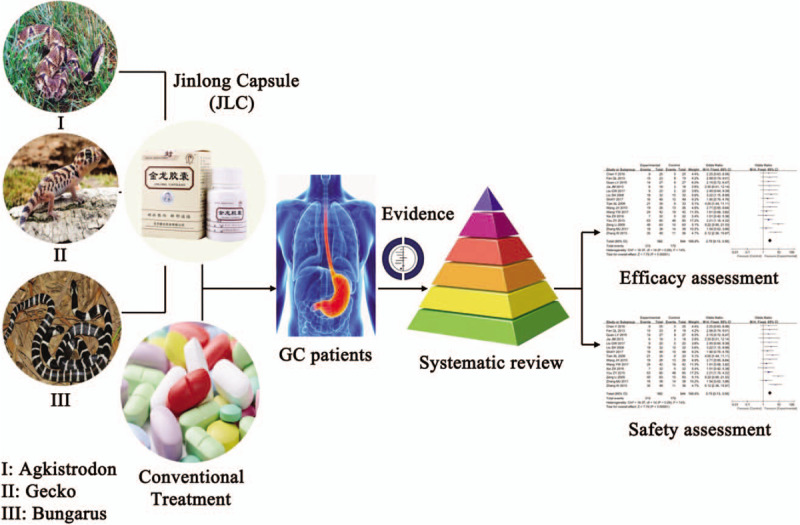
Work flow of the present study.

Many studies have reported that the addition of JLC could be improve GC patients’ survival time, quality of life (QoL), immune function and reduce side effects caused by radiochemotherapy.^[13,17]^ Despite the intensive clinical studies, its clinical efficacy was still not well established and recognized. We are prepared to summarize the efficacy and adverse events of JLC treatment of GC at advanced stages through the meta-analysis, in order to provide scientific reference for the design of future clinical trials (Fig. 1).

## 2 Study aim

The aim of this meta-analysis was to systematically evaluate the efficacy and safety of JLC mediated therapy for the treatment of advanced GC.

## 3 Methods

The protocol of our meta-analysis will be reported according to Preferred Reporting Items for Systematic Review and Meta-Analysis Protocols (PRISMA-P) guidelines.^[19]^ Our protocol has been registered on the International Platform of Registered Systematic Review and Meta-Analysis Protocols (INPLASY). The registration number was INPLASY202040105 (DOI number is 10.37766/inplasy2020.4.0105). This meta-analysis is a secondary research which based on some previously published data. Therefore, the ethical approval or informed consent was not required in this study.

### 3.1 Dissemination plans

We will disseminate the results of this systematic review by publishing the manuscript in a peer-reviewed journal or presenting the findings at a relevant conference.

### 3.2 Eligibility criteria

#### 3.2.1 Study designs to be included

All available randomized controlled trials (RCTs) that investigated the efficacy and safety of JLC-mediated therapy in patients diagnosed with advanced GC will be included in this systematic review. Articles without sufficient available data, noncomparative studies, non-RCTs, literature reviews, meta-analysis, meeting abstracts, and case reports will be excluded.

#### 3.2.2 Participant or population

Patients must be cytologically or pathologically confirmed as having GC at a clinically advanced stage. There will be no limitations on age, gender, racial, and region. Patients with other malignancies or non-primary GC are not included.

#### 3.2.3 Intervention

In the experimental group, advanced GC patients must be treated with conventional treatment (including chemotherapy, radiotherapy, and targeted therapy) combined with JLC mediated therapy.

#### 3.2.4 Comparator

In the control group, GC patient treated with the same conventional treatment as intervention group in the same original study.

#### 3.2.5 Types of outcome

##### 3.2.5.1 Main outcome(s)

The primary outcomes will be the therapeutic effects of treatment according to Response Evaluation Criteria in Solid Tumors 1.1 (RECIST Criteria 1.1).^[20]^

(a) Overall response rate and disease control rate;
(b) Overall survival (which is defined as the time from the date of randomization to death from any cause);
(c) Disease-free survival (which is the time from date of random assignment to date of recurrence or death).

##### 3.2.5.2 Additional outcome(s)

Secondary outcomes will include:

1. immune function evaluation;
2. QoL as evaluated by Karnofsky score, and
3. treatment–related adverse effects assessment.

### 3.3 Information sources

Eight electronic databases including Cochrane Library, PubMed, Web of Science (WOS), Excerpt Medica Database (Embase), Chinese Biomedical Literature Database (CBM), China National Knowledge Infrastructure (CNKI), China Scientific Journal Database (VIP), and Wanfang Database will be systematically searched for eligible studies from their inception to January 2020. Language is limited with English and Chinese.

### 3.4 Search strategy

To perform a comprehensive and focused search, experienced systematic review researchers will be invited to develop a search strategy. The plan searched terms are as follows: “gastric cancers” or “gastric neoplasm” or “gastric carcinoma” or “gastric tumor” or “stomach cancers” or “stomach neoplasm” or “stomach carcinoma” or “stomach tumor” or “GC” or “SC” and “Jinlong Capsule” or “JLC” et al. An example of search strategy for PubMed database shown in Table 1 will be modified and used for the other databases.

**Table 1:**
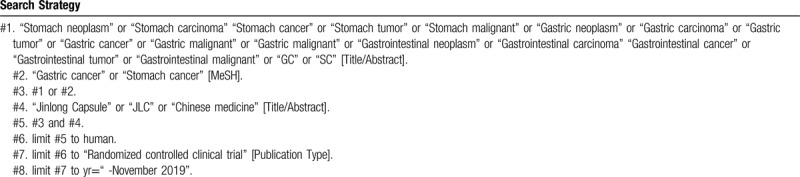
Searching strategy in PubMed.

### 3.5 Study selection and management

**Figure 2:**
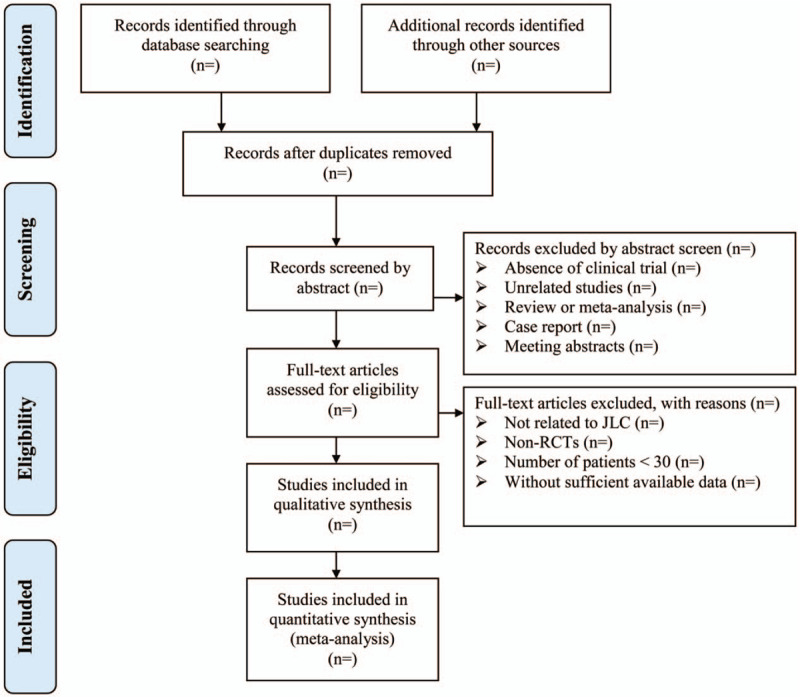
Study selection process for the meta-analysis. JLC = Jinlong capsule, RCTs = randomized controlled trials.

Endnote X7 software will be used for literature managing and records searching. Two authors (Li Jianwei Li and Bin Han) will be reviewed independently to identify potential trials by assessing the titles and abstracts. The full text of all relevant trials will be further evaluated to make sure eligible trials. Any conflict will be resolved through discussion with a third reviewer. A PRISMA-compliant flow diagram (Fig. 2) will be used to describe the selection process of eligible literatures.

### 3.6 Data extraction and management

Two reviewers (Bin Han and Guangzong Sun) will be responsible for the data extraction independently according to the Cochrane Handbook for Systematic Reviews of Intervention. The following data will be extracted from eligible literatures: the first author, year of publication, country of study, participants (sample size, TNM stage, age, gender, inclusion and exclusion criteria, etc), details of all experimental and control interventions regimen (manufacturer of the drugs, dosage of JLC, administration route, duration of treatment, follow-up time, etc), outcomes (overall response rate, disease control rate, overall survival, disease-free survival, QoL, immune function and adverse effects). For survival outcomes, Hazard ratios with corresponding 95% confidence intervals will be extracted from trials or be estimated from Kaplan–Meier survival curves by established methods.^[21]^ Any disagreements will be resolved by discussion, and a third reviewer (Zhong Zheng) will make the final decision. Excluded studies and the reasons for exclusion will be listed in a table.

### 3.7 Dealing with missing data

We will attempt to contact the authors to request the missing or incomplete data. If those relevant data are not acquired, they will be excluded from the analysis.

### 3.8 Quality assessment/risk of bias analysis

The quality of the included RCTs will be assessed independently by 2 investigators (Jianwei Li and Bin Han) in terms of sequence generation, allocation concealment, blinding, incomplete outcome data, selective reporting, and other bias, according to the guidance of the Cochrane Handbook for Systematic Review of Interventions.^[22,23]^ Evidence quality will be classified as low risk, high risk, or unclear risk of bias in accordance with the criteria of the risk of bias judgment. Any disagreements will be resolved via discussion with a third researcher (Guangzong Sun).

### 3.9 Strategy of data synthesis

Statistical analyses will be performed using Review Manager 5.3 (Nordic Cochran Centre, Copenhagen, Denmark) and Stata 14.0 (Stata Corp, College Station, TX) statistical software. The outcomes were mainly represented by risk ratio with its 95% confidence intervals. A 2-tailed *P* value <.05 was considered statistically significant. Cochrane *Q*-test and *I*^2^ statistics were used to assess heterogeneity between studies; *P* < .1 or *I*^2^ > 50% indicates statistical heterogeneity.^[24]^ A fixed effect model will be used to calculate the outcomes when statistical heterogeneity is absent; otherwise, the random effects model was considered according to the DerSimonian and Laird method.^[25]^

### 3.10 Publication bias analyses

We will detect publication biases and poor methodological quality of small studies using funnel plots if 10 or more studies are included in the meta-analysis. Begg and Egger regression test will be utilized to detect the funnel plot asymmetry.^[26–28]^ If publication bias existed, a trim-and-fill method should be applied to coordinate the estimates from unpublished studies, and the adjusted results were compared with the original pooled risk ratio.^[29,30]^

### 3.11 Assessment of heterogeneity

#### 3.11.1 Sensitivity analysis

Sensitivity analysis was conducted to explore an individual study's influence on the pooled results by deleting 1 single study each time from pooled analysis. A summary table will report the results of the sensitivity analyses.

#### 3.11.2 Subgroup and meta-regression analysis

If the data are available and sufficient, subgroup and meta-regression analysis will be conducted to explore the source of heterogeneity with respect to age, gender, region, tumor stage, sample sizes, follow-up period, chemotherapy regimens, and types of involved studies.

## 4 Discussion

Traditional Chinese medicine has an extensive history and has been widely used as an effective adjuvant drug for cancer treatment in China, Japan, and other Asian countries.^[5]^ In addition to focusing on the survival rate of cancer patients, how to improve the QoL of cancer patients has also become important. Traditional Chinese interventions have huge advantages in treating cancer, enhancing immunity, and improving the QoL of cancer patients.^[31–35]^ As an effective Chinese medicine preparation, preclinical studies indicate that JLC can inhibit proliferation, migration, and invasion of tumor cells, and induces apoptosis in a variety of cancer cells.

### 4.1 Strengths and limitations of this study

Even though there was statistical analysis of published clinical trials, the exact therapeutic effects of JLC mediated therapy were still not systematically investigated. This systematic review may provide helpful evidence for clinicians, and patients who use JLC preparations for the treatment of advanced GC.

The systematic review will also have some limitations. There may be a language bias with the limitation of English and Chinese studies. Clinical heterogeneity may exist for different tumor stage and ages of GC patients, conventional treatment regimens, dosage of JLC, and duration of treatment.

## Author contributions

Jingxia Chi and Jianwei Li conceived the concept and designed the study protocol. Jianwei Li, Bin Han and Guangzong Sun tested the feasibility of the study. Jianwei Li, Bin Han, Guangzong Sun and Zhong Zheng wrote the manuscript. Jingxia Chi, Jianwei Li, Bin Han and Ying Mu provided methodological advice, polished and revised the manuscript. All authors approved the final version of the manuscript.

**Conceptualization:** Jianwei Li, Jingxia Chi.

**Funding acquisition:** Ying Mu.

**Investigation:** Jianwei Li, Bin Han, Guangzong Sun, Jingxia Chi.

**Methodology:** Jianwei Li, Bin Han, Ying Mu, Jingxia Chi.

**Project administration:** Jianwei Li, Jingxia Chi.

**Supervision:** Jianwei Li, Jingxia Chi.

**Writing –original draft:** Jianwei Li, Bin Han, Guangzong Sun, Zhong Zheng, Jingxia Chi.

**Writing –review & editing:** Jianwei Li, Bin Han, Ying Mu, Jingxia Chi.
